# Rac1 and Rac3 GTPases differently influence the morphological maturation of dendritic spines in hippocampal neurons

**DOI:** 10.1371/journal.pone.0220496

**Published:** 2019-08-01

**Authors:** Roberta Pennucci, Irene Gucciardi, Ivan de Curtis

**Affiliations:** San Raffaele—Vita-Salute University and San Raffaele Scientific institute, Cell Adhesion Unit, Division of Neuroscience, Milano, Italy; Universita degli Studi di Torino, ITALY

## Abstract

The Rac1 and Rac3 GTPases are co-expressed in the developing nervous system, where they are involved in different aspects of neuronal development, including the formation of synapses. The deletion of both Rac genes determines a stronger reduction of dendritic spines *in vitro* compared to the knockout of either gene, indicating that Rac1 and Rac3 play a synergistic role in the formation of these structures. Here, we have addressed the role of each GTPase in the formation of dendritic spines by overexpressing either Rac1 or Rac3 in wildtype neurons, or by re-expressing either GTPase in double knockout hippocampal cultures. We show that the Rac3 protein is expressed with Rac1 in developing hippocampal neurons. Overexpression of either GTPase in WT neurons increases the density of dendritic spines, suggesting the involvement of both GTPases in their formation. We also found that the re-expression of either Rac1 or Rac3 in double knockout neurons is sufficient to restore spinogenesis. Rac1 is significantly more efficient than Rac3 in restoring the formation of spines. On the other hand the quantitative analysis in neurons overexpressing or re-expressing either GTPase shows that Rac3 induces a more pronounced increase in the size of the spines compared to Rac1. These enlarged spines form morphological synapses identified by the juxtaposition of postsynaptic and presynaptic markers. Thus, while Rac1 appears more efficient in inducing the formation of mature spines, Rac3 is more efficient in promoting their enlargement. Our study highlights specific roles of Rac1 and Rac3, which may be functionally relevant also to synaptic plasticity.

## Introduction

The actin cytoskeleton plays a central role in the formation and function of postsynaptic dendritic spines [1–6], where it is necessary for the organization of the postsynaptic densities [7] and anchoring of postsynaptic receptors [8]. Defects in the regulation of the actin cytoskeleton may cause mental disability linked to memory deficits [9]. Distinct pools of F-actin regulate in a dynamic way the structure and the plasticity of the dendritic spines [10], indicating that the actin–mediated structural changes in the spines play a crucial role in synaptic plasticity and in learning and memory [11].

The Rac proteins belong to the Rho family of small GTPases, and are central organizers of the actin cytoskeleton. Of the three highly homologous Rac GTPases expressed in vertebrates, Rac1 is ubiquitously expressed [12], and it is co-expressed with Rac3 in the developing nervous system [13,14]. Rac1 and Rac3 are involved in different aspects of neuronal development including neuronal migration, neurite extension and branching, and synaptogenesis [15–17]. Moreover, the alteration of the regulation and function of these proteins is linked to cognitive impairment [18,19].

The deletion of the two genes for Rac1 and Rac3 in mice affects the development of specific populations of cortical neurons [20–22]. *In vitro* the double deletion of Rac1 and Rac3 strongly impairs the development of spines in cultured hippocampal neurons, and the weaker phenotypes observed in neurons with single Rac deletion indicate that either GTPase may partially compensate for the lack of the other Rac protein during the development of dendritic spines [20].

Rac3 plays distinct functions *in vitro* compared to Rac1 in developing retinal neuron and in neuroblastoma cells [23,24]. To address Rac3 function *in vivo*, Rac3 knockout (KO) mice were generated. These mice do not show evident anatomical defects in the organization of the brain [25], nor evident defects are seen in cultured hippocampal neurons in terms of neuronal polarity, neuritogenesis and formation of synaptic contacts [26]. On the other hand, although Rac1 may partially compensate for the function of Rac3 in the Rac3 KO animals, these mice have interesting behavioral alterations characterized by a hyperactive and hyper-reactive behavior to new stimuli [27], which suggest a cognitive impairment induced by the deletion of this gene.

Moreover, it is intriguing that the KO of both Rac genes *in vitro* determines a strong reduction of dendritic spines and a strong increase in dendritic filopodia [20]. This effect is considerably stronger than the defect induced by KO of only Rac1, indicating that Rac3 is also required for spine development, and that Rac1 and Rac3 play a synergistic function in the formation of dendritic spines. Here we have addressed the contribution of the two GTPases by overexpressing *in vitro* either Rac in wildtype (WT) hippocampal neurons, and by re-expressing either Rac1 or Rac3 in cultured double KO hippocampal neurons.

## Materials and methods

### Antibodies

Antibodies (Abs) used for immunoblotting were: anti-Rac1 mAb (BD Biosciences, 610651); rabbit anti-Rac3 pAb [25]; horseradish peroxidase (HRP)-conjugated sheep pAb anti-mouse IgG (GE Healthcare, NA931); HRP-conjugated donkey pAb anti-rabbit IgG (GE Healthcare, NA934). Abs used for immunofluorescence were: rabbit pAb anti-GFP (Invitrogen, A11122); chicken pAb anti-GFP (Abcam, ab13970); goat pAb anti-PAK3 (Santa Cruz); mAb anti-Cre (Covance, MMS-106P); rabbit pAb anti-Homer (Synaptic Systems, 160003); mouse mAb anti-VAMP2 (Synaptic Systems, 104201). Secondary Abs: goat A488 pAb anti-rabbit IgG (Invitrogen, A11008); goat A488 pAb anti-chicken IgG (Invitrogen, A11039); donkey A568 pAb anti-rabbit IgG (Invitrogen, A10042); goat A647 pAb anti-mouse IgG (Invitrogen, A31571); goat A568 pAb anti-mouse IgG (Invitrogen, A11004).

### Plasmids

The following plasmids were utilized: pEGFP-N1 (Clontech, Mountain View, CA), pEGFP-Cre [20]; pEGFP-Rac1 e pEGFP-Rac3 were obtained by subcloning the cDNAs for Rac1 and Rac3 into pEGFP-C1 (Clontech).

### Mice

Mice were held in the animal house at our Institute. All procedures for experiments with mice were carried out according to the guidelines at the San Raffaele Scientific Institute, in agreement with the national (D.L. n 116, G.U. suppl. 40, February 18 1992, circular Nr. 8, G.U., July 14 1994) and international rules (EEC Council directive 86/609, OJ L 358, 1 DEC 12, 1987). The animal research ethics committee at the San Raffaele Institute (Comitato Istituzionale per la buona sperimentazione animale, Ospedale San Raffaele) approved the use of mice to set up primary cultures of hippocampal neurons for this research (approval SK 616). Mice were sacrificed by CO_2_ inhalation and cervical dislocation.

We used C57BL/6J wild type (WT; from Harlan Laboratories) and Rac3 KO mice [25] to obtain primary cultures of hippocampal neurons. Rac1F/F//Rac3KO mice (with floxed Rac1 gene, [20]) were used to generate hippocampal cultures with double Rac1/Rac3 KO, that could be obtained by transfecting the Rac1F/F//Rac3KO neurons with a plasmid for the Cre recombinase. Mice were genotyped by PCR on genomic DNA from mice tails, as described [20].

### Hippocampal cultures and transfection

Neuronal cultures were prepared from hippocampi of embryonic day 17.5 (E17.5) mice (WT or Rac1F/F//Rac3KO), as described [28]. Hippocampi were resuspended in 4.5 ml of Hank's balanced salt solution (HBSS) added with 500 μl of trypsin (Invitrogen, 15090–046) and 25 μl of DNAse (Sigma-Aldrich). After 14 min incubation at 37°C, trypsinization was stopped by adding 7 ml of plating medium (Neurobasal medium, 10% fetal bovine serum, 2% B27 supplements, 1% GlutaMAX). After two washes with plating medium, the pellet was resuspended in 2–3 ml of plating medium, hippocampi were mechanically dissociated, and plated onto poly-L-lysine–coated glass coverslips (10^5^ cells per coverslip). For biochemical analysis, 1.3 x 10^6^ neurons were plated in 6 ml of plating medium in a 60 mm diameter dish. After incubation for 3 h at 37°C and 5% CO_2_, maintenance medium (Neurobasal medium, 2% B27 supplements 1%, GlutaMAX 1%) was added to each dish. Neurons were incubated at 37°C in a 5%CO_2_ humidified atmosphere for the times indicated. For longer culture times, every 7 days in vitro one third of the volume of medium was replaced by fresh maintenance medium.

DIV4 hippocampal neurons were either transfected with pEGFP-N1 plasmid, or cotransfected with pEGFP-N1 and pEGFP-CRE [29] by using Lipofectamine 2000 (Invitrogen) in maintenance medium. Transfection of the GFP-Cre has been used to show the effects of the deletion of endogenous proteins on the maturation of hippocampal neurons *in vitro* [20;29]. Neurons were cultured up to DIV14. After fixation with 4% paraformaldehyde in 4% sucrose, 2 mM EGTA, 120 mM sodium phosphate (pH 7.4), cells were permeabilized with 0.3% Triton X-100 and processed for immunofluorescence with the indicated antibodies. Primary antibodies were detected with Alexa Fluor 488/568-conjugated secondary antibodies (Molecular Probes). Images were captured with a Zeiss Axiophot epifluorescence microscope equipped with a C4742-95-12HR digital camera (Hamamatsu, Hamamatsu City, Japan).

### Biochemical analysis

Hippocampal cultures and P7 brains were extracted at 4°C with lysis buffer [0.5% Triton-X-100, 1 mM NaV, 10 mM NaF, antiprotease mix Complete (Roche, Manheim, Germany) in TBS]. For immunoprecipitation, anti-Rac3 pAb pre-adsorbed to 25 μl of protein A-Sepharose beads (Amersham Biosciences, Piscataway, NJ) was added to aliquots of lysates (1 mg protein) and incubated with rotation for 2 h at 4°C. For control, aliquots of lysates were incubated with uncoated beads, or with beads incubated with preimmune serum. Immunoprecipitates were washed 3 times with 1 ml of washing buffer with 0.5% Triton X-100. Immunoprecipitates, lysates and unbound fractions were analyzed by immunoblotting.

### Immunofluorescence and quantitative analysis

DIV14 hippocampal neurons were fixed for 15 min at RT in 4% paraformaldehyde, 4.1% sucrose, 3 mM EGTA, 2 mM MgCl_2_, in phosphate buffered saline (PBS). Neurons were incubated overnight at 4°C with the indicated Abs diluted in GSDB (goat serum dilution buffer: 0.3% Triton X-100, 16% goat serum, 450 mM NaCl, 20 mM sodium phosphate, pH 7.4). Neurons were incubated with secondary Abs conjugated to Alexa Fluor A488/568/647 and 4',6-diamidin-2-fenilindolo (DAPI) diluted in GSDB. Washed coverslips were mounted with 70% glycerol and 0.01% phenylethylendiammine in PBS.

Samples were observed with a microscope Olympus IX-70 equipped with a CoolSnap HQ CCD camera, and with a deconvolution system DeltaVision RT (Applied Precision’s, Inc.). When indicated, image stacks along the Z axis were deconvoluted, and analyzed by the ImageJ software (NIH). Confocal analysis was performed with a Leica TCP SP2 (Leica Microsystems). Images were processed and analyzed by using the Volocity 3D Image Analysis software (PerkinElmer).

The density of dendritic protrusions and the area of the spine heads were calculated by the Adobe Photoshop 8 and ImageJ software. Two independent experiments for each condition were analyzed. The data were analyzed by the one-way analysis of variance (ANOVA) followed by the Bonferroni post hoc test for significance. Differences were considered statistically significant for *P* < 0.05. The analysis of the effects on the density of the dendritic protrusions was evaluated by analyzing secondary and tertiary dendritic segments. For each segment, protrusions were counted and classified on the base of their morphology, to distinguish between the mature spines including the mushroom, thin and stubby subtypes, and the immature spines including short (< 4 μm) and long (> 4 μm) filopodia, as well as lamellipodia. Dendritic protrusions were identified on the basis of their morphological features as follows: thin spines with slender neck and a small head, mushroom spines with a larger head and a usually relatively short neck, stubby spines with no neck, and filopodia with no head. The number of protrusions was normalized to dendritic segment lengths of 50 μm.

The area of the heads of mushroom and thin spines was measured by the ImageJ software.

### Live time-lapse imaging

Transfected neurons were monitored by time-lapse imaging using a UltraVIEWERS Spinning Disk confocal microscope (Perkin Elmer), equipped with a 63x lens and an OkoLab chamber to control temperature and humidity. For each time-point, Z-stack of sections 6 to 9 μm thick were acquired to generate a Z-projection image. Images were processed using the Volocity 3D Image Analysis software (PerkinElmer).

## Results and discussion

### Expression of Rac3 in cultured hippocampal neurons

**Fig 1:**
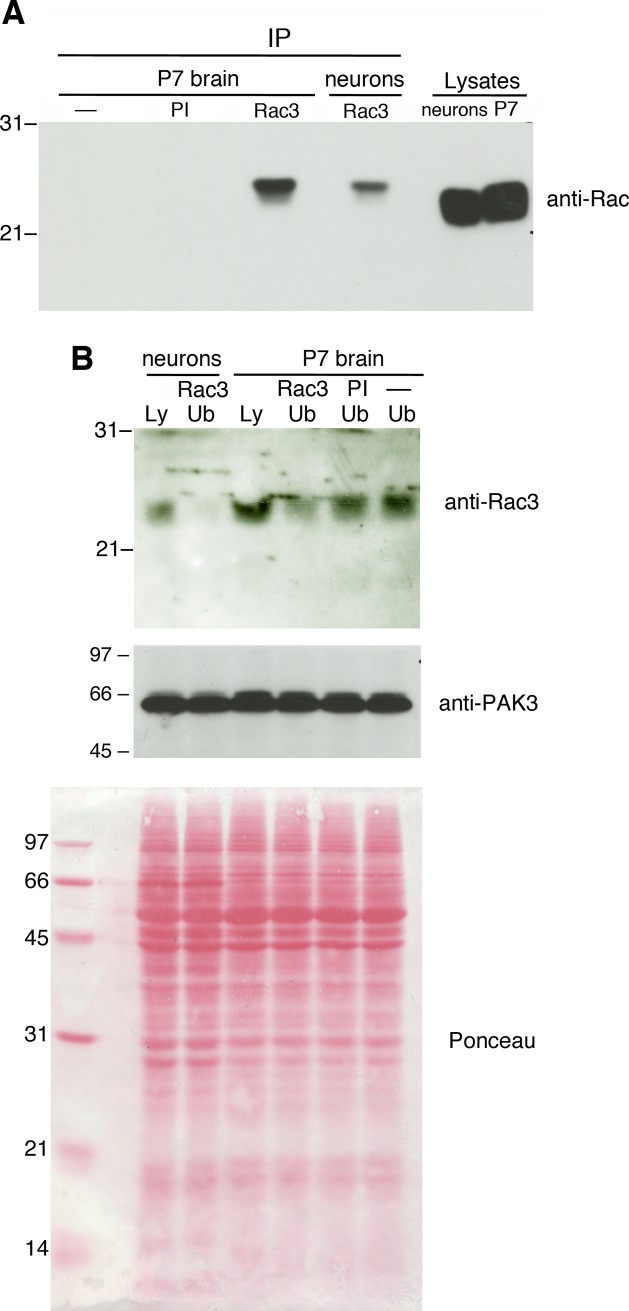
Rac3 expression in DIV14 hippocampal cultures. **(A)** Aliquots of lysates (1 mg protein lysate / IP) from either P7 mouse brain or from DIV14 mouse hippocampal cultures were used for immunoprecipitation by incubation with Protein A sepharose beads alone (–) bound to preimmune serum (PI) or to anti-Rac3 pAb (Rac3), or to preimmune serum (PI). Immunoprecipitates were immunoblotted with anti-Rac mAb recognizing both Rac1 and Rac3. **(B)** Lysates (Ly, 100 μg/lane) and unbound fractions after immunoprecipitation (Ub, 100 μg/lane) with beads bound to anti-Rac3 pAb (Rac3), preimmune (PI), or with beads alone (–) were blotted with the anti-Rac3 pAb (top filter) or with anti-PAK3 goat pAb (center). Below is the filter stained with Ponceau.

Rac1 and Rac3 GTPases are co-expressed in the mammalian nervous system. While Rac1 is ubiquitously expressed during neuronal development and in the adult, Rac3 has a more limited expression during neuronal development, reaching a peak of expression during the period of intense neurite branching and synaptogenesis [23,30]. Previous studies also from our laboratory have shown a synergy between Rac1 and Rac3, both *in vivo* and *in vitro*, which is required for the correct development of a functional nervous system [20,22]. Analysis on hippocampal cultures has shown that spinogenesis is strongly inhibited especially after removal of both GTPases, suggesting functional cooperation between Rac1 and Rac3 in this process [20]. On the other hand, the more limited pattern of expression of Rac3 suggests that this GTPase may play a specific role in the regulation of the correct synaptic development. Here we have addressed the function of each Rac GTPase in the formation of dendritic spines *in vitro*, by using the well established hippocampal cultures, which recapitulate the different phases of neuronal development in a simplified setting. We have immunoprecipitated Rac3 from lysates of mouse hippocampal cultures maintained for 14 days in vitro (DIV14), using lysates from postnatal day 7 (P7) mouse brain as control, since at this age Rac3 protein expression is highest in the mouse brain [30]. Immunoprecipitation was done with an antibody specific for Rac3. Immunoprecipitates were then analyzed by immunoblotting with an anti-Rac mAb recognizing both Rac1 and Rac3 (**Fig 1A**). The strong band recognized by the anti-Rac mAb in the lysates indicates that quantitatively Rac3 represents a minor fraction of the total Rac proteins present in these neurons, consistent with what had been previously reported for the whole developing mouse brain [20]. The specificity of the immunoprecipitation was confirmed by the absence of signal when lysates were incubated with beads alone or bound to preimmune serum. We used anti-Rac rather than anti-Rac3 to blot the immunoprecipitates, since the use of anti-Rac3 for blotting on filters containing Rac3 IgG used for the immunoprecipitations gives a strong background obscuring the detection of immunoprecipitated Rac3. On the other hand, lysates and unbound fractions after immunoprecipitation were immunoblotted with the anti-Rac3 Ab showing the specific depletion of Rac3 in the unbound fraction after immunoprecipitation with anti-Rac3 (**Fig 1B**). Therefore the hippocampal neurons at DIV14 express Rac3. Rac GTPase play crucial roles in distinct aspects of neuronal development [4,15]. Analysis of the conditional deletion of Rac1 in developing neurons and full Rac3 knockout have shown that these GTPases play essential function during late neuronal and cerebral development [20,22,31]. Knockout of both Rac *in vitro* determines a significant reduction of total dendritic protrusions that include spines and filopodia: neurons show a strong reduction in mature spines, while filopodia increase [20]. To determine the contribution of each GTPase in the formation of spines we have tested the effects on dendritic protrusion density and dimension of the spine heads after overexpression or re-expression of either Rac1 or Rac3 in mouse cultured hippocampal neurons from WT or KO mice, respectively.

### Overexpression of either Rac1 or Rac3 increases the density of dendritic spines and the size of mature spines

To determine the effects of the overexpression of either Rac on the development of dendritic spines, hippocampal neurons from WT mice were transfected with plasmids for GFP (control), GFP-Rac1 or GFP-Rac3.

**Fig 2:**
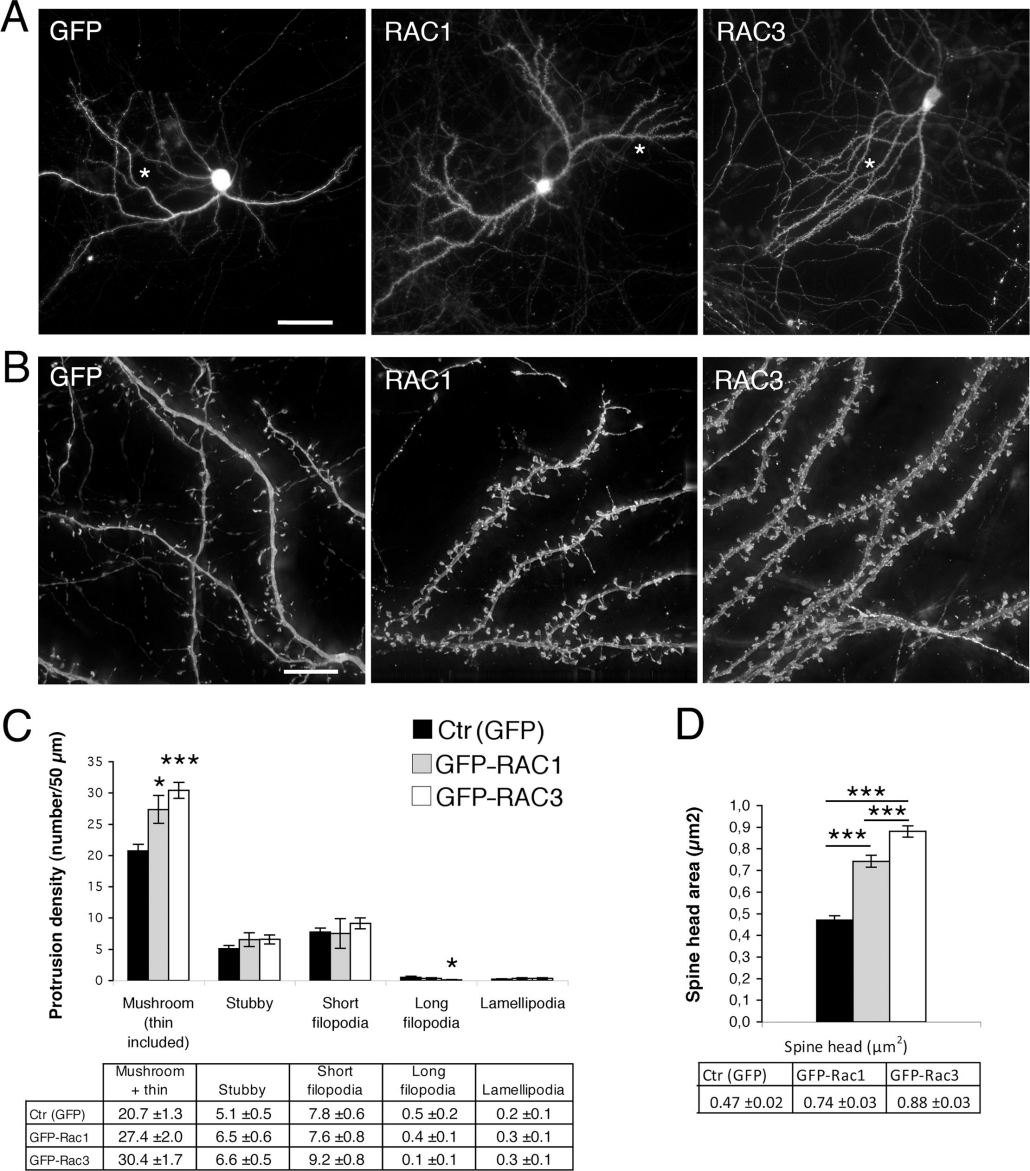
The overexpression of Rac GTPases increases the density and size of dendritic spines. (**A**) Morphology of DIV14 hippocampal neurons transfected with GFP, GFP-Rac1, or GFP-Rac3. Hippocampal neurons from WT mice were transfected at DIV4, fixed at DIV14, and stained with anti-GFP Abs. Bar, 50 μm. (**B**) Higher magnifications of WT hippocampal neurons transfected as in (**A**). Bar, 10 μm. (**C**) Quantification of the density of dendritic protrusions expressed as the mean density of mature spines, long (> 4 μm) and short (< 4 μm) filopodia, and lamellipodia (number/50 μm dendrite). Mature spines include mushroom, thin and stubby subtypes. Quantification was performed on 58–66 dendritic segments from 12–17 neurons per experimental condition. Data are presented as means ±SEM. *P < 0.05; ***P < 0.001 (one-way ANOVA with Bonferroni post hoc test). (**D**) Quantification of the area of the head of mature spines (mushroom e thin). Quantification was performed on 210–251 spines from 9–14 neurons per experimental condition. Data are presented as means ±SEM. *** P < 0.001 vs GFP–transfected cells (one-way ANOVA with Bonferroni post hoc test).

Morphological analysis on transfected DIV14 WT hippocampal neurons (**Fig 2A**) indicated that the overexpression of either Rac1 or Rac3 induced a similar increase in the density of dendritic protrusions with respect to control neurons transfected with GFP (**Fig 2B**). Both Rac1 and Rac3 induced an increase in mushroom and thin spines (**Fig 2C**): we observed a 32% increase in Rac1–overexpressing neurons (*P* < 0.01 for Rac1– vs GFP–transfected cells), and a 47% increase in Rac3–overexpressing neurons (*P* < 0.001 for Rac3– vs GFP–transfected neurons). Only mild differences were observed for stubby spines and long filopodia only in Rac3-overexpressing neurons, while short filopodia and lamellipodia were not affected by Rac overexpression.

The quantitative analysis performed on morphologically mature spines in neurons overexpressing either GTPase suggested an increase in the size of the spines compared to control GFP–positive cells (**Fig 2B**). The area of the head of the morphologically mature spines was measured for each experimental condition (**Fig 2D**). The overexpression of Rac1 determined a significant increase of the head area (+58%, *P* < 0.001 vs GFP). Interestingly, Rac3 overexpression led to a significantly stronger increase in the area of the spine heads with respect to Rac1 overexpression (+87%, *P* < 0.001 vs GFP; +19%, *P* < 0.001 vs Rac1. **Fig 2D**).

### Neurons overexpressing Rac form spines that carry synapses

**Fig 3:**
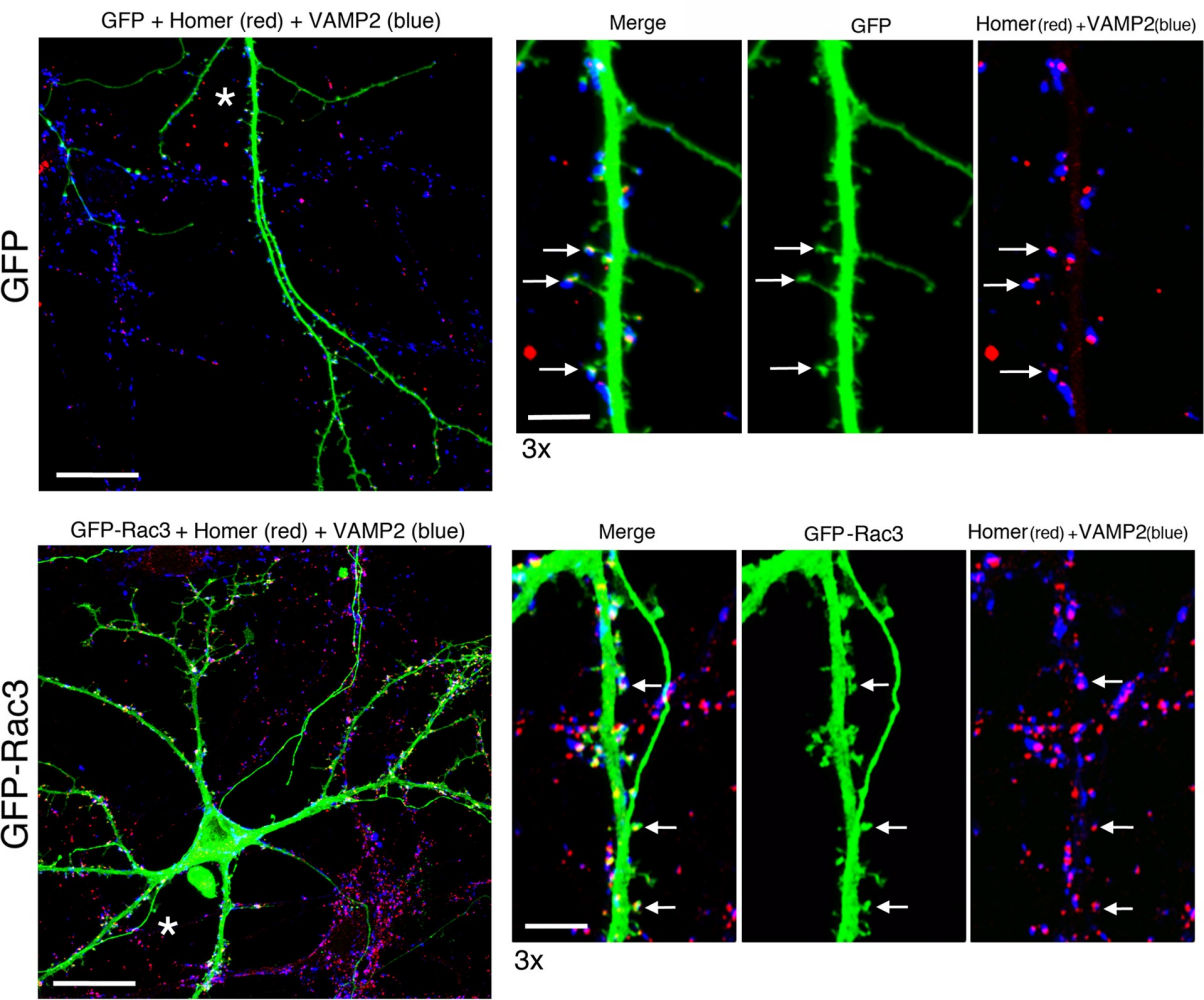
Morphological synapses in GFP-Rac3 positive hippocampal neurons. WT mouse hippocampal neurons transfected at DIV4 with plasmids for GFP (upper panels) or GFP-Rac3 (lower panels) were fixed at DIV14 and immunostained with antibodies specific for GFP (green), presynaptic marker VAMP2 (blue), and postsynaptic marker Homer (red). On the right are shown enlargements of the dendritic branches indicated with asterisks in the correspoinding low magnification images (left panels). Puncta with evident juxtaposition between VAMP2 and Homer (arrows show examples) are considered putative morphologically mature synapses. Bars, 20 μm (left panels) and 5 μm (right panels).

To verify if the morphologically larger mature spines forming in cells overexpressing Rac3 are potentially functional, we evaluated their correspondence with synaptic contacts. For this, transfected DIV14 hippocampal neurons were immunolabelled with Abs specific for proteins of the synaptic terminals: the postsynaptic marker Homer, and the presynaptic marker VAMP2. Homer gave a punctate labelling along the dendrites of transfected neurons. Often the Homer-positive puncta showed evident juxtaposition with presynaptic VAMP2–positive puncta, indicating the presence of putative synapses. Most spines observed in control (GFP) and GFP-Rac3 overexpressing neurons represent potential synaptic sites, including the spines showing larger heads observed in neurons overexpressing Rac3 (**Fig 3**): quantification showed that 91% of the spines examined in neurons transfected with GFP (n = 235 spines from 8 dendritic branches) and 96% of the spines examined in neurons transfected with GFP-Rac3 (n = 265 spines from 9 dendritic branches) carried synapses, as detected by the juxtaposition of the immunostaining for the postsynaptic marker Homer and the presynaptic marker VAMP2.

### The re-expression of either Rac1 or Rac3 in double KO neurons affects differently the formation of dendritic spines

**Fig 4:**
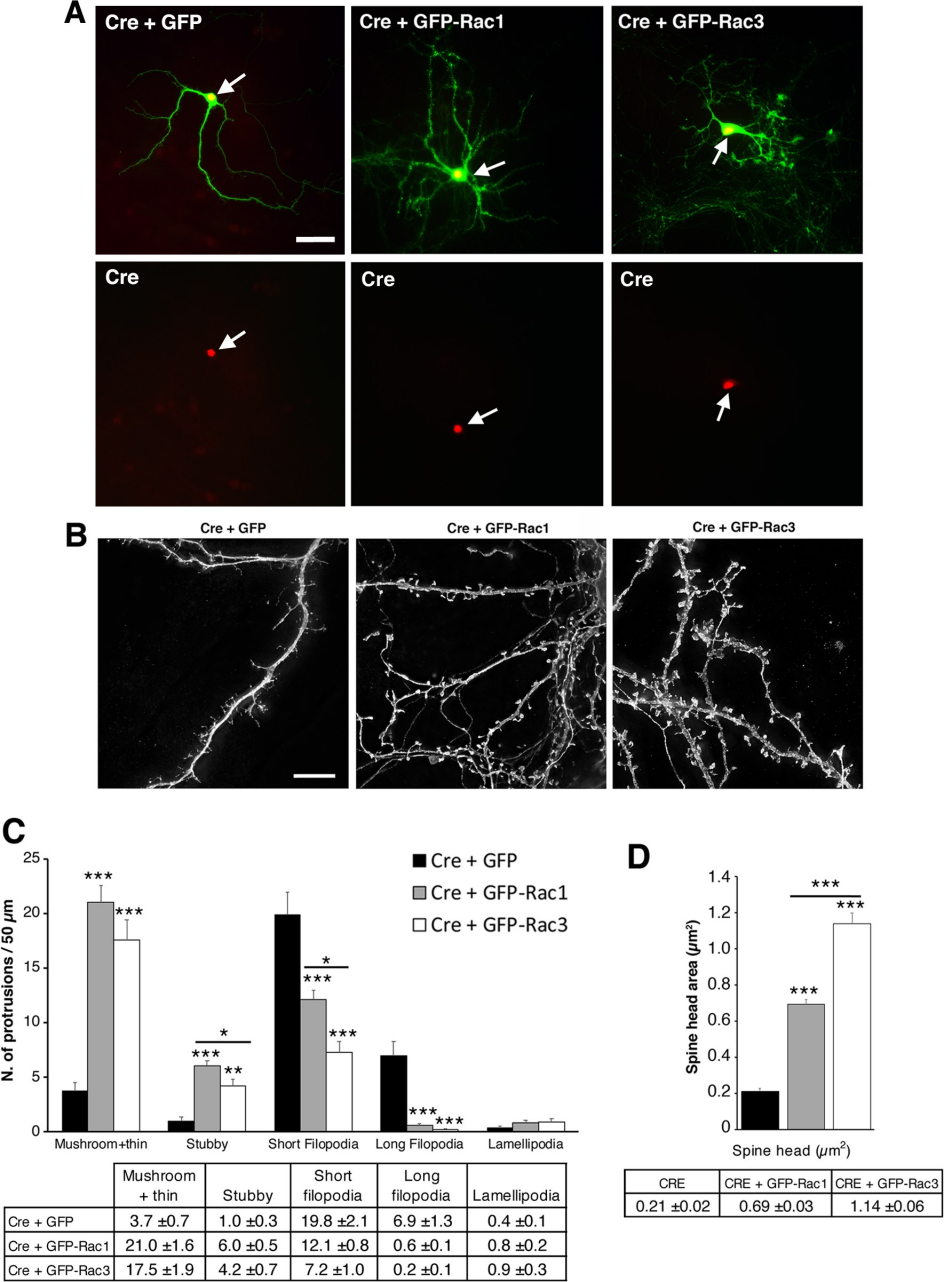
The re-expression of either Rac1 or Rac3 GTPase in double KO neurons differently affects the formation of dendritic spines. (**A**) Morphology of DIV14 Rac3 KO (*Rac1F/F//Rac3KO*) hippocampal neurons cotransfected at DIV4 with the plasmid for the Cre recombinase together with the plasmid for either GFP, GFP-Rac1, or GFP-Rac3. Neurons were fixed at DIV14 and stained with anti-GFP Ab (green) and anti-Cre Ab (red, nuclear). Images at the bottom show the same neurons from the top row, to highlight the nuclear localization of the Cre recombinase. Bar, 50 μm. (**B**) High magnifications of double KO hippocampal neurons (*Rac1F/F//Rac3KO* neurons transfected with Cre recombinase as in **A**). Bar, 10 μm. (**C-D**) DIV14 hippocampal neurons treated as in (**A-B**) were used to quantify the density and size of mature spines. (**C**) Density of dendritic protrusions including mature spines, filopodia, and lamellipodia. Bars represent the number of protrusions / 50 μm of dendritic branch. Mature spines include both mushroom and thin types quantified together, as well as stubby spines. Bars are means ±SEM (n = 22–49 dendritic segments from 8–11 neurons/experimental condition). *P < 0.05, **P < 0.01, ***P < 0.001 (one-way ANOVA with Bonferroni post hoc test). (**D**) Quantification of the area of mushroom spine heads: n = 54 spines (Cre + GFP); 241 spines (Cre + GFP-Rac1); 137 spines (Cre + GFP-Rac3). Bars are means ±SEM. ***P < 0.001 (one-way ANOVA with Bonferroni post hoc test).

We next compared the re-expression of either Rac1 or Rac3 in hippocampal neurons carrying the double deletion of Rac1 and Rac3 GTPases. To generate cultures with the double KO of these GTPases, we induced the deletion of Rac1 *in vitro* by expressing in hippocampal neurons from *Rac1F/F//Rac3 KO* mice a plasmid for the expression of the Cre recombinase. For this, *Rac1F/F//Rac3KO* hippocampal cultures were cotransfected with a plasmid for the Cre together with a plasmid for either GFP (control), GFP-Rac1, or GFP-Rac3. In this way, both the deletion of the endogenous genes for Rac1 and Rac3, and the re-expression of either GFP-tagged GTPase could be achieved. The expression of the transfected Cre was evaluated for each neuron included in the analysis (**Fig 4A**). As previously shown by this laboratory [20], the deletion of both genes for Rac1 and Rac3 strongly inhibited the formation of dendritic spines (**Fig 4B**). Re-expression of either Rac in the double KO neurons significantly increased the spinogenesis (**Fig 4B and 4C**). Interestingly, the re-expression of either Rac GTPase also significantly reduced the density of filopodia along dendrites, suggesting that the abundant filopodia present in the double KO neurons may represent precursors of developing spines that could not mature in the absence of the Rac GTPases. Interestingly, the increased density of dendritic filopodia is also a hallmark of impaired neuronal maturation and Rac dysregulation, as observed for example in *Fmr1* KO mice [32]; the alteration of Rac function may lead to altered Rac-mediated cytoskeletal rearrangements that cause defects in neuronal development, including reduced dendritic branching, as observed for example in *ArhGAP15* KO mice [33].

The mean density of total dendritic protrusions was increased significantly in double KO neurons expressing GFP-Rac1 (protrusions/50 μm dendritic length: 40.7 ±1.8 SEM; n = 49), compared to double KO neurons expressing either GFP (protrusions/50 μm dendritic length: 31.8 ±3.0 SEM; n = 22; *P* = 0.0162), or GFP-Rac3 (protrusions/50 μm dendritic length: 30.0 ±2.68 SEM; n = 26; *P* = 0.0015). On the other hand, no significant difference in total dendritic protrusions was observed between double KO neurons expressing GFP-Rac3 and double KO neurons expressing GFP (*P* = 0.6494). Moreover, there were dramatic changes in the different types of protrusions forming after re-expression of either GTPase. In Rac re-expressing neurons there was a strong increase in mushroom spine density (5.7-fold for Rac1 and 4.7-fold for Rac3; *P* < 0.001) and stubby spines (6,3-fold for Rac1, and 4.3-fold for Rac3; *P* < 0.001) compared to double KO neurons (**Fig 4C**). On the other hand the density of filopodia was strongly reduced compared to double KO cells in cells re-expressing either Rac1 (–53%) or Rac3 (–73%).

The re-expression of either GTPase significantly and strongly increased the size of mushroom spines, as detected by measuring the area of their heads (**Fig 4D**): Rac1 and Rac3 expression induced a 3.3-fold (*P* < 0.001) and 5.4-fold (*P* < 0.001) increase in average spine head area respectively, compared to the average spine head area for the few spines formed by the double KO neurons. Interestingly, as already observed after overexpressing either GTPase in the WT neurons, Rac3 has a stronger effect compared to Rac1 on the size of the spines. In the case of re-expression of the Rac3 GTPase in double KO cells the effects were more evident compared to its overexpression in WT cells that express endogenous Rac3. The increase of the mean spine head area induced by Rac3 was 1.6-fold that induced by the re-expression of Rac1 (*P* < 0.001). Altogether these data indicate that the re-expression of either Rac1 or Rac3 was sufficient to re-establish the formation of dendritic spines, and that Rac3 promoted the formation of larger spines.

## Conclusions

The formation and modulation of synaptic contact sites is at the basis of cerebral activity, and requires the formation of spines as the post-synaptic counterpart of terminal axonal endings. Rac1 and Rac3 are co-expressed at time of synaptogenesis during brain development [15]. Studies on murine KO models for both or either GTPase have shown that Rac1 and Rac3 play essential and synergistic roles during late neuronal development [20,22,31]. In particular Rac3 is mainly expressed during late development [13,14], with a peak of protein expression in the mouse brain at times of major neurite branching and synaptogenesis [30]. The more restricted spatial and temporal patterns of expression of Rac3 compared to Rac1 suggest that this GTPase may exert a specific role during synaptic development. The present study has shown that the Rac3 protein is expressed with Rac1 in DIV14 hippocampal neurons. Overexpression of either GTPase in WT neurons induced an increase in the density of spines along dendrites, suggesting the involvement of both GTPases in the formation and maturation of the spines.

The double KO neurons were characterized by an elevated density of filopodia and strongly decreased spines compared to WT neurons. Re-expression of either Rac1 or Rac3 was sufficient to significantly improve spinogenesis, with a corresponding decrease in filopodia that may represent immature spine precursors [34–36]. These data suggest that Rac1 and Rac3 are not required for the formation of filopodial precursors, while they are needed for the later maturation of filopodial precursors into spines. To be noted that the re-expression of Rac1 appeared more efficient than Rac3 in supporting the formation of spines, since Rac1 re-expression induced a significantly higher increase of stubby spines and a nonsignificant higher increase of mushroom spines. Stubby spines have been shown to decrease in number during development, and they have been proposed to represent precursors that would then outgrow and elongate into mature thin and mushroom spines [37]. These data indicate that the two Rac proteins appear to play at least a partially redundant role in the process of spine formation.

On the other hand, the analysis of the effects of the overexpression or re-expression of either Rac protein on the size of mature spines has highlighted significant differences between Rac1 and Rac3. A previous study showed an increase in spine size upon expression of Rac3 in rat cortical neurons [38]. Here, comparative quantitative analysis on neurons overexpressing either Rac1 or Rac3 protein has shown that although the overexpression of either Rac induced a significant increase in the area of the mature spines, the increase was significantly more pronounced after overexpression of Rac3. The enlarged spines formed upon Rac3 overexpression form morphological synapses that are potentially functional, since in most cases they include postsynaptic densities as detected by immunostaining with postsynaptic markers, and they are juxtaposed to presynaptic terminals identified by specific markers. The difference of the effects on the size of the spine head was even stronger when either Rac was re-expressed in neurons depleted of both endogenous proteins, indicating that Rac3 plays a prominent role in the regulation of the spine size. The enlargement of the spine head is a morphological event associated to synaptic potentiation [10]. During evolution the gene for Rac3 has appeared in the vertebrates [15], suggesting a possible specific role of this GTPase in the regulation of cognitive functions. This hypothesis is supported by recent findings that mutations of this gene in humans cause severe forms of intellectual disability [39].

Thus, while Rac1 appears more efficient in inducing the formation of mature spines, Rac3 is more efficient in promoting their enlargement. Since the enlargement of the head of the spines is a morphological event observed in synapses undergoing LTP, our results suggest that Rac3 may be involved in the structural potentiation of synapses, an intriguing hypothesis that will need to be explored in the future.

Our study highlights a specific role of Rac3 in the organization of dendritic spines, and future studies will be necessary to test whether the morphological differences observed in this study are functionally relevant in synaptic function and plasticity. This analysis will be relevant also to the comprehension of the mechanisms underlying human intellectual disability that is caused by recently identified point mutations of the gene for Rac3 [39].
